# Acetylsalicylic acid rescues the immunomodulation of inflamed gingiva-derived mesenchymal stem cells via upregulating FasL in mice

**DOI:** 10.1186/s13287-019-1485-5

**Published:** 2019-12-03

**Authors:** Tingting Yu, Boxi Yan, Jing Li, Ting Zhang, Ruili Yang, Xuedong Wang, Yan Liu, Dawei Liu

**Affiliations:** 1 Department of Orthodontics, National Engineering Laboratory for Digital and Material Technology of Stomatology, Beijing Key Laboratory of Digital Stomatology, Peking University School and Hospital of Stomatology, Beijing, People’s Republic of China; 2 Second Clinical Division, Peking University School and Hospital of Stomatology, Beijing, People’s Republic of China

**Keywords:** Mesenchymal stem cell, Immunomodulation, Cell therapy, Fas pathway

## Abstract

**Background:**

Gingiva-derived mesenchymal stem cells (GMSCs) obtained multipotent differentiation and immunomodulatory properties. However, collecting healthy gingival tissues may be challenging in the clinical situation. Thus, in our present study, we aim to evaluate whether the immunomodulatory capacity of gingiva-derived mesenchymal stem cells from inflamed gingival tissues (iGMSCs) is impaired and find a way to rescue their deficient properties.

**Methods:**

We compared the immunomodulation capacity of GMSCs and iGMSCs using an in vitro co-culture system and a mouse colitis model. T cell apoptosis, T helper 17 (Th17), and regulatory T (Treg) cell differentiation were detected by flow cytometry analysis.

**Results:**

We demonstrated that iGMSCs obtained a decreased immunomodulatory capacity compared with GMSCs. Acetylsalicylic acid (ASA) pretreatment was able to rescue iGMSCs’ impaired immunomodulatory properties. Mechanistically, ASA was capable of upregulating the expression of Fas ligand (FasL) in iGMSCs, leading to an improvement in iGMSC-mediated T cell apoptosis and therapeutic efficacy in the treatment in colitis mice.

**Conclusions:**

This study indicates that the deficient immunomodulatory function of iGMSCs could be rescued by ASA pretreatment via upregulating of FasL in mice*.* This strategy might serve as a practical approach to rescue deficient MSC function for further therapeutic application.

## Background

Dental tissue-derived mesenchymal stem cells (MSCs) are becoming attractive options for their easy accessibility, low immunogenicity, and considerable therapeutic potential [1]. Dental tissue-derived MSCs are originated from the neural crest, which obtain superior stemness compared with bone marrow mesenchymal stem cells (BMMSCs) [2]. Gingiva-derived mesenchymal stem cells (GMSCs) are isolated from gingiva tissues, which can form colonies, express MSC surface markers, and have the ability to differentiate into osteogenic, adipogenic, and chondrogenic tissues [3–7]. Importantly, GMSCs are readily accessible from the oral cavity and can also be easily found in discarded tissue samples in clinic. Previous studies suggested that GMSCs could be applied in cell therapy clinically, such as MSC-based bone regeneration [8, 9]. Furthermore, previous studies also revealed that GMSCs are capable of regulating immune responses [8].

However, since 5–20% of the population are suffering from gingivitis or periodontitis [10], collecting a considerable amount of healthy gingiva tissue to isolate usable GMSCs becomes challenging. Thus, it is interesting to know whether immunomodulation properties are impaired in GMSCs derived from inflammatory tissues (iGMSCs), such as in gingivitis or periodontitis. Moreover, if we can rescue the immunomodulation properties of iGMSCs by a simple treatment without genetic modification, it will be of great treatment potential to facilitate cell-based therapy.

In this study, we revealed that not only stemness but also the immunomodulation capacity of iGMSCs was impaired, including decreased ability to induce T cell apoptosis and less curative effect on colitis mice. Interestingly, when treated with acetylsalicylic acid (ASA), a kind of widely used nonsteroidal anti-inflammatory drug (NSAID), the deficient immunomodulatory capacity of iGMSCs was rescued both in vitro and in vivo. Mechanistically, we showed that ASA could upregulate the FasL expression of iGMSCs to induce activated T cell apoptosis and rescue their immunomodulation function.

## Methods

### Animals and cell culture

Male C57BL/6J mice (Weitong Lihua, China) were used for the animal experiment, which was performed under the protocol for the use of animal research approved by Peking University (LA2013-92).

Periodontitis was induced by silk ligature as previously reported [11]. Mouse gingival tissues from the maxillary molar region were separated, cut up, and digested in phosphate-buffered saline (PBS) containing 4 mg/mL Dispase II (Roche Diagnostics, USA) and 2 mg/mL collagenase type I (Worthington Biochemical, USA) at 37 °C for 1 h. Gingival tissues from three individual mice were pooled together trying to minimize heterogeneity. By passing cells through a 70-μm strainer, we obtained single-cell suspensions. All nucleated cells (ANCs) were seeded at 1 × 10^6^ with α-MEM (Invitrogen, Carlsbad, CA, USA) supplemented with 20% FBS, 55 μM 2-mercaptoethanol (Invitrogen), 2 mM l-glutamine (Invitrogen), 100 U/mL penicillin, and 100 μg/mL streptomycin (Invitrogen). The ANCs were initially incubated for 48 h at 37 °C and 5% CO_2_. We washed the cultures with PBS to eliminate the nonadherent cells. Then, under the same conditions, the attached cells were cultured for another 12 days as in the complete medium.

### Antibodies and reagents

Fas ligand (FasL) antibodies (SC-33716, Santa Cruz Biotechnology, USA) and anti-β-actin antibody (A1978, Sigma-Aldrich, USA) were used. Allophycocyanin (APC)-conjugated anti-IFN-γ, phycoerythrin (PE)-conjugated anti-IL-17, APC-conjugated anti-CD3, APC-conjugated anti-CD25, and peridinin chlorophyll protein complex (Percp)-conjugated anti-CD4 antibodies were purchased from eBioscience. Anti-CD45, CD73, CD90, CD105, and CD146 conjugated with PE and anti-CD34 and STRO1 conjugated with FTIC were purchased from BD Biosciences (Franklin Lakes, USA). BrdU solution and BrdU imaging kit were purchased from Invitrogen (Carlsbad, CA, USA).

### CFU-F assay

Isolated independent ANCs (1 × 10^5^) from the gingiva were seeded into 60-mm culturing plates (Corning). The plates were stained with a solution of 2% paraformaldehyde (PFA; Merck, Germany) and 0.1% toluidine blue (Merck, Germany) after 14 days of culturing. A single colony cluster was defined as colonies that contained more than 50 cells. Colony-forming unit-fibroblastic (CFU-F) counting was performed in five independent samples per experimental group.

### Flow cytometry analysis

SCA1-PE, CD105-PE, CD90-PE, CD44-PE, CD73-PE, CD45-PE, CD34-PE, CD4-Percp, CD3-APC, and CD25-APC antibodies were used for surface staining. Foxp3-PE and IL-17-PE antibodies were used for intracellular staining. Cells were analyzed with a flow cytometer (BD Biosciences, USA).

### Cell proliferation assay

GMSCs and iGMSCs (1 × 10^4^/well) were seeded on chamber slides (Nunc, USA). Cultures were then incubated with BrdU solution (1:100; Invitrogen) for 20 h and stained with a BrdU staining kit (Invitrogen) according to the manufacturer’s instructions. We used ten representative images to calculate the number of cells with BrdU-positive nuclei to quantify cell proliferation capacity. The percentage of BrdU-positive cells in the total cell number was calculated. Three independent samples of each experimental group were used for BrdU assay.

### Population doubling

Multiple single-colony-derived GMSCs were trypsinized and seeded in 35-mm dishes (Corning) at 2 × 10^5^ in complete growth medium for passage one. When confluence was reached, cells were harvested and seeded at the same number. The following formula was used for population doubling (PD): PD = log2 (number of harvested cells/number of seeded cells). The cumulative addition of total cell numbers generated from each passage until ceasing dividing was determined as the PD numbers. For each group, the PD assay was repeated with three independent isolated cells.

### T lymphocyte apoptosis assay

Stem cells (0.2 × 10^6^/per well) were seeded into a 24-well culture plate (Corning) which contained Dulbecco’s modified Eagle’s medium (DMEM; Switzerland) with 10% fetal bovine serum (FBS), 50 μM 2-mercaptoethanol, 1 mM sodium pyruvate (Sigma-Aldrich), 10 mM HEPES, 1% nonessential amino acid (Cambrex, USA), 100 U/mL penicillin, 100 mg/mL streptomycin, and 2 mM l-glutamine. After 24-h incubation, T lymphocytes (1 × 10^6^) from the spleen were treated with plate-coated anti-CD3ε (3 μg/mL) and anti-CD28 (2 μg/mL) antibodies, which were then co-cultured with stem cells for 2 days. CD3 antibody staining was used to detect apoptotic T cells, which was followed by the Annexin V Apoptosis Detection Kit (BD Biosciences, USA) detection.

### Western blot

Total protein was extracted using M-PER Mammalian Protein Extraction Reagent (Thermo, USA). Twenty micrograms of protein was applied and separated on 4 to 12% NuPAGE gel (Invitrogen), which was then transferred to nitrocellulose membranes (Millipore, USA). The membranes were blocked with 5% nonfat dry milk and 0.1% Tween-20 for 1 h, which were then incubated with the primary antibodies at 4 °C overnight. We used HRP-conjugated secondary antibody (1:10,000; Santa Cruz Biotechnology) to treat the membranes for 1 h. Using SuperSignal West Pico Chemiluminescent Substrate (Thermo) and BioMax film (Kodak, USA), immunoreactive proteins were detected. To quantify the amount of loaded protein, each membrane was stripped using stripping buffer (Thermo) and incubated with anti-β-actin antibody.

### Dextran sulfate sodium-induced mouse colitis and treatment with GMSCs or iGMSCs

By administration of 3% (*w*/*v*) dextran sulfate sodium (DSS; MP Biochemicals, USA) to drinking water for 10 days, acute colitis was induced in 8-week-old C57BL/6 mice [8]. On day 3 post-DSS induction, 1 × 10^6^ stem cells were injected into colitis mice through the tail vein (one sample for all treated mice in each group). All mice were sacrificed at day 10 for further examination as previously described [12]. We used PBS to suspend GMSCs or iGMSCs for injection; the colitis mice without MSC treatment were also treated with 200 μl PBS (WT group).

Colitis severity was assessed by body weight loss and histologic activity index (HAI), which was based on hematoxylin and eosin (HE) staining of the sections (*n* = 5 in each group). HAI parameters included infiltration and epithelial damage. The HAI score was finally calculated as the sum of the infiltration and epithelium score, ranging from 0 (unaffected) to 8 (severe colitis).

### FasL knockdown

We seeded 2 × 10^5^ GMSCs into a 12-well culture plate with the presence of FasL siRNA (SC-29313, Santa Cruz Biotechnology, USA) following the manufacturer’s protocols to knockdown FasL expression.

### Statistical analysis

For two-group comparisons, we used independent two-tailed Student’s *t* tests for statistical analysis (SPSS 13.0, USA). One-way analysis of variance (ANOVA) was used for comparisons between more than two groups followed by the least significant difference (LSD) multiple-comparison test. Statistical significance was considered when *p* values were less than 0.05.

## Results

### iGMSCs obtained impaired stemness compared with GMSCs

**Fig. 1:**
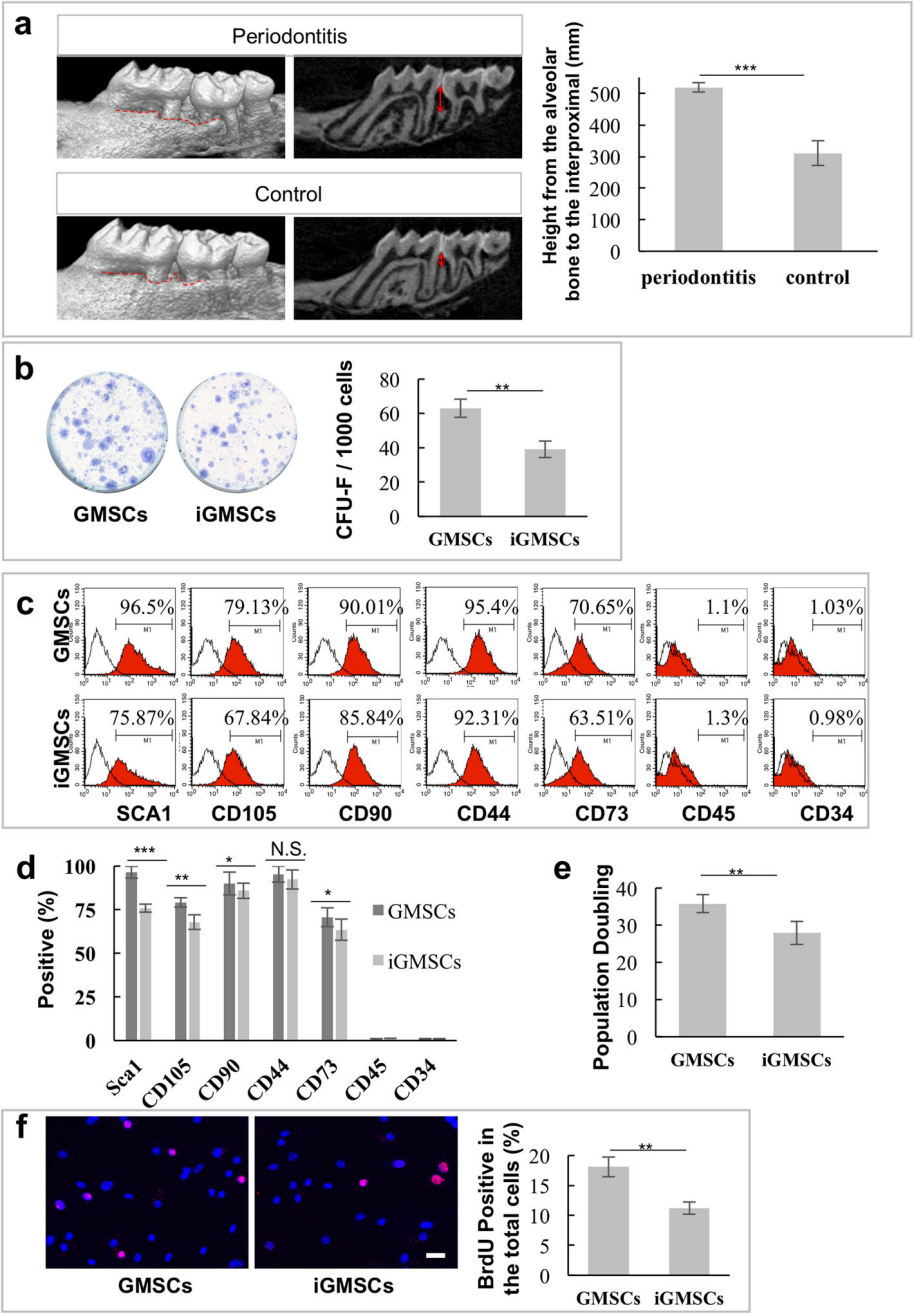
iGMSCs obtained impaired stemness compared with GMSCs. **a** Micro-CT showed significant alveolar bone resorption in ligature-induced periodontitis in mice. **b** iGMSCs exhibited reduced single colony-forming ability. **c**, **d** Flow cytometric analysis of the expression of Sca1, CD105, CD90, CD73, CD44, CD45, and CD34 in iGMSCs and GMSCs. **e** Continued culture assay showed that iGMSCs had less elevated population doubling than GMSCs. **f** BrdU^+^ labeling assay showed decreased proliferation rate in iGMSCs compared with GMSCs. *n* = 5 in each group. *P < 0.05. **P < 0.01. ***P < 0.005. Error bars: mean ± SD

To determine whether iGMSCs maintain the characteristics of MSCs in inflammatory microenvironment, we isolated iGMSCs from the gingiva tissues of periodontitis mice. The mice with periodontitis we used were evaluated with significant alveolar bone resorption as detected by micro-CT (Fig. 1a).

We found that iGMSCs exhibited reduced single colony-forming ability as assessed by CFU-F assays (Fig. 1b). Flow cytometric analysis showed that MSC surface markers including Sca1, CD105, CD90, and CD73 significantly decreased in iGMSCs, while CD45 and CD34, hematopoietic lineage markers, were absent in both iGMSCs and GMSCs (Fig. 1c, d). Moreover, we also found a second population from iGMSCs displayed in the forward versus side scatter flow cytometry dot plots (Additional file 1: Figure S1), which indicated that the inflammation factors might direct iGMSCs differentiated into a second cell population. We then demonstrated that iGMSCs showed a decreased proliferation rate, as determined by population doubling (Fig. 1e) and BrdU^+^ labeling assay (Fig. 1f), when compared with GMSCs. Moreover, the differentiation potential toward the osteocyte, adipocyte, and chondrocyte of iGMSCs decreased, as assessed by alizarin red, oil red O, and toluidine blue staining (Additional file 1: Figure S2a-c), respectively. These data indicated that the stemness of iGMSCs was impaired.

### iGMSCs showed impaired immunomodulatory properties

**Fig. 2:**
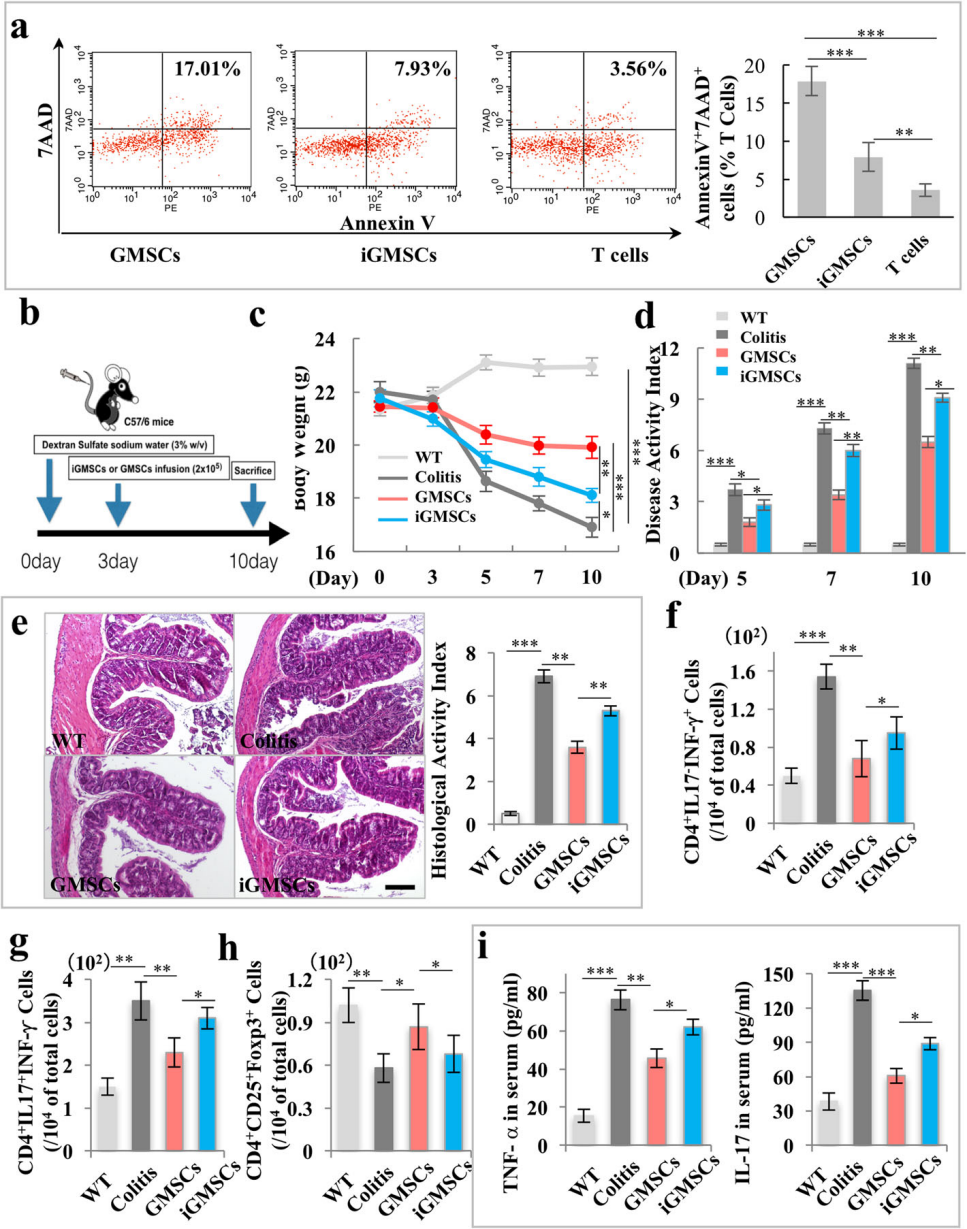
The immunomodulatory properties of iGMSCs are impaired. **a** In vitro co-culture system showed a significantly decreased capacity of iGMSCs to induce Annexin V^+^/7AAD^+^ T cell apoptosis when compared with the GMSC group. **b** Schema showing iGMSC and GMSC transplantation for treating dextran sodium sulfate (DSS)-induced experimental colitis mice. **c**–**e** iGMSCs showed impaired immunomodulation capacity compared with GMSCs, as assessed by **c** amelioration of losing body weight, **d** decreased disease activity index (DAI), and **e** alleviation of colitis histologic activity index (HAI). **f**–**h** FACS analysis showed that the levels of Th1 and Th17 were significantly elevated while the levels of Tregs were significantly reduced in colitis mice compared with the control mice. iGMSC infusion exhibited compromised reduction of Th1 and Th17 cells and upregulation of Treg levels in colitis in mice than did GMSCs. **i** ELISA analysis showed that the levels of tumor necrosis factor α (TNF-α) and IL-17 in serum were markedly increased in colitis mice compared with the control mice at 10 days post-DSS induction. iGMSC infusion exhibited compromised ability to downregulate serum levels of TNF-α and IL-17 compared with GMSCs. *n* = 5 in each group. Scale bar = 200 μm. *P < 0.05. **P < 0.01. ***P < 0.005. Error bars: mean ± SD

Since previous studies have suggested that GMSCs possessed immunomodulatory properties [3, 8], we next examined whether the inflammatory condition could influence the GMSCs’ immunomodulatory capacity. We first used a GMSCs/T cell or iGMSCs/T cell co-culture system to examine its immunomodulation effects in vitro*.* Flow cytometric analysis revealed that iGMSCs showed significant reduced capacity to induce both the early (Annexin V^+^/7AAD^−^ cells) and later (Annexin V^+^/7AAD^+^ cells) T cell apoptosis (Fig. 2a and Additional file 1: Figure S3). Then, we used experimental colitis mice to evaluate the immunomodulatory properties of iGMSCs in vivo. GMSCs or iGMSCs (1 × 10^6^) were intravenously infused into experimental colitis mice on day 3 (Fig. 2b). A significant decreased body weight was detected among colitis mice when compared with the control C57BL/6J mice; the infusion of iGMSCs was not able to restore the reduced body weight as effectively as that of GMSCs (Fig. 2c). Furthermore, iGMSC treatment could not effectively rescue the disease activity index (DAI), including body weight loss, bleeding, and diarrhea, compared with the GMSC infusion group (Fig. 2d). Histologically, iGMSCs also failed to eliminate inflammatory cells and recover the epithelial structure compared with the GMSC group, as evaluated by the histological activity index including suppressing epithelial ulceration, ameliorating colonic transmural inflammation, restoring normal intestinal architecture, and reducing wall thickness (Fig. 2e). Moreover, flow cytometric analysis revealed that, compared to the GMSC group, iGMSCs showed significant reduced capacity to downregulate the elevated level of CD4^+^ IFN-γ^+^ T helper 1 (Th1) and CD4^+^ IL-17^+^ (Th17) cells and reduced level of CD4^+^ CD25^+^ Foxp3^+^ regulatory T cells (Tregs) in colitis mice at day 10 post-DSS induction (Fig. 2f–h). Meanwhile, iGMSCs were not able to induce as effective reductions of serum levels of TNF-α and IL-17 in colitis mice as did the GMSCs, assessed by enzyme-linked immunosorbent assay (ELISA) (Fig. 2i). Taken together, these data indicated that iGMSCs had impaired immunomodulatory properties.

### ASA treatment rescues the deficient immunomodulatory properties of iGMSCs

**Fig. 3:**
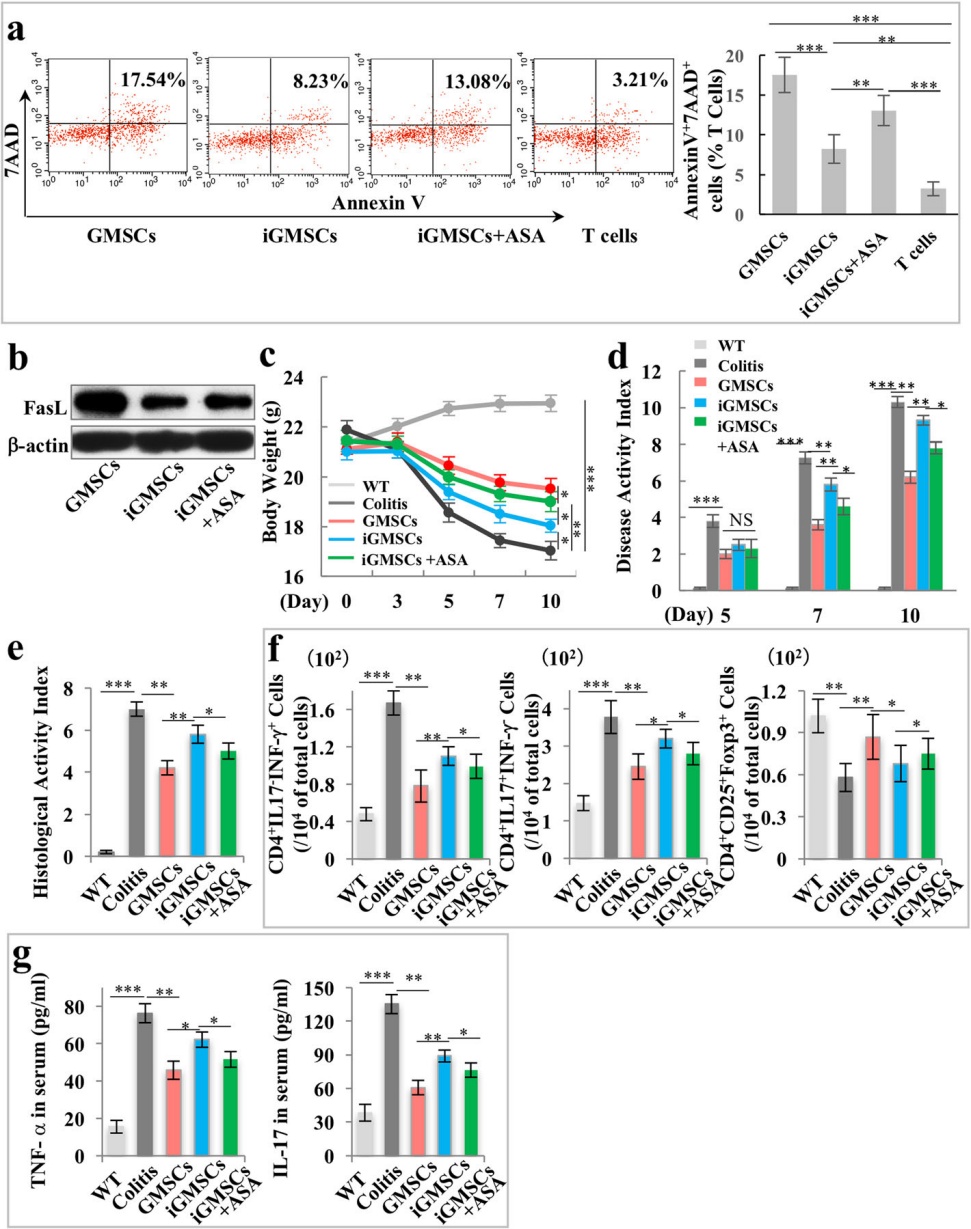
ASA treatment rescues the impaired immunomodulatory properties of iGMSCs. **a** In vitro co-culture system showed a significantly increased capacity of ASA-treated iGMSCs to induce Annexin V^+^/7AAD^+^ T cell apoptosis when compared with the iGMSC group. **b** Western blot analysis showed that ASA treatment elevated the expression of FasL in iGMSCs. **c**–**e** ASA-treated iGMSCs showed restored immunomodulation capacity compared with untreated iGMSCs, as assessed by **c** amelioration of losing body weight, **d** decreased disease activity index (DAI), and **e** alleviation of colitis histologic activity index (HAI). **f** FACS analysis showed that ASA-treated iGMSC infusion rescued the iGMSCs’ ability to reduce Th1 and Th17 cells and elevate Treg cells in colitis mice. **g** ELISA analysis showed that ASA-treated iGMSC infusion rescued the iGMSCs’ ability to downregulate the levels of TNF-α and IL-17 in colitis mice. *n* = 5 in each group. Scale bar = 200 μm. *P < 0.05. **P < 0.01. ***P < 0.005. Error bars: mean ± SD

Since the immunomodulatory effects of GMSCs were impaired in inflammatory microenvironment, we next sought to rescue their affected immunomodulation function in vitro for further application. ASA-pretreated stem cells from human exfoliated deciduous teeth (SHED) and BMMSCs were previously reported to improve bone regeneration and immunomodulatory properties [13–15]; therefore, we tested whether it could rescue the deficient immunomodulatory function of iGMSCs. Interestingly, flow cytometric analysis showed that ASA-treated iGMSCs (ASA-iGMSCs) obtained a significantly increased capacity to induce both the early (Annexin V^+^/7AAD^−^ cells) and later (Annexin V^+^/7AAD^+^ cells) T cell apoptosis (Fig. 3a and Additional file 1: Figure S4a).

We next sought to explore the underlying mechanism of ASA rescuing the immunomodulation properties of iGMSCs. Since the Fas/FasL-mediated cell death pathway represented a typical apoptotic signaling in various cell types [16, 17], we hypothesized that ASA may be able to rescue the immunomodulation of iGMSCs by elevating FasL activity. Western blot analysis confirmed that the expression of FasL in iGMSCs was low compared to that in GMSCs, and ASA pretreatment was able to elevate FasL expression (Fig. 3b and Additional file 1: Figure S4b). However, as other immunosuppressive molecules are also critical for inducing retard T cell proliferation and enhancing apoptosis, we evaluated the expression levels of iNOS, TGF-β, and PEG6 by qPCR and found that iGMSCs displayed a decreased expression profile compared with GMSCs (Additional file 1: Figure S4c); the results indicated that these molecules might also participate in the immunomodulatory capacity of GMSCs. Next, to further confirm if the deficiency of FasL in iGMSCs leads to low immunomodulation capacity, we used siRNA to knockdown the FasL expression level in GMSCs (Additional file 1: Figure S4d); the co-culture system demonstrated that siFasL-treated GMSCs exhibited a decreased capacity to induce activated T cell apoptosis when compared with GMSCs (Additional file 1: Figure S4e). Then, to evaluate if ASA treatment was able to rescue the immunomodulation capacity of iGMSCs, we used ASA-iGMSCs to treat colitis mice in vivo; ASA-iGMSCs showed significantly improved restoration of reduced body weight and DAI score in colitis mice when compared with the iGMSC group from 7 to 10 days (Fig. 3c, d). Also, the histologic activity index (HAI) indicated that ASA-iGMSCs had superior ability to eliminate inflammatory cells and recover the epithelial structure (Fig. 3e). ASA-iGMSC treatment obtained a better therapeutic effect in downregulating the levels of Th1 and Th17, as well as upregulating the level of Tregs, compared with iGMSCs (Fig. 3f). Also, ASA-iGMSCs induced more significant reductions in serum TNF-α and IL-17 level in colitis mice compared with the iGMSC group (Fig. 3g). These results suggested that ASA was capable of recovering the immunomodulation capacity of iGMSCs.

### ASA treatment rescues the impaired immunomodulation of iGMSCs via upregulating FasL

**Fig. 4:**
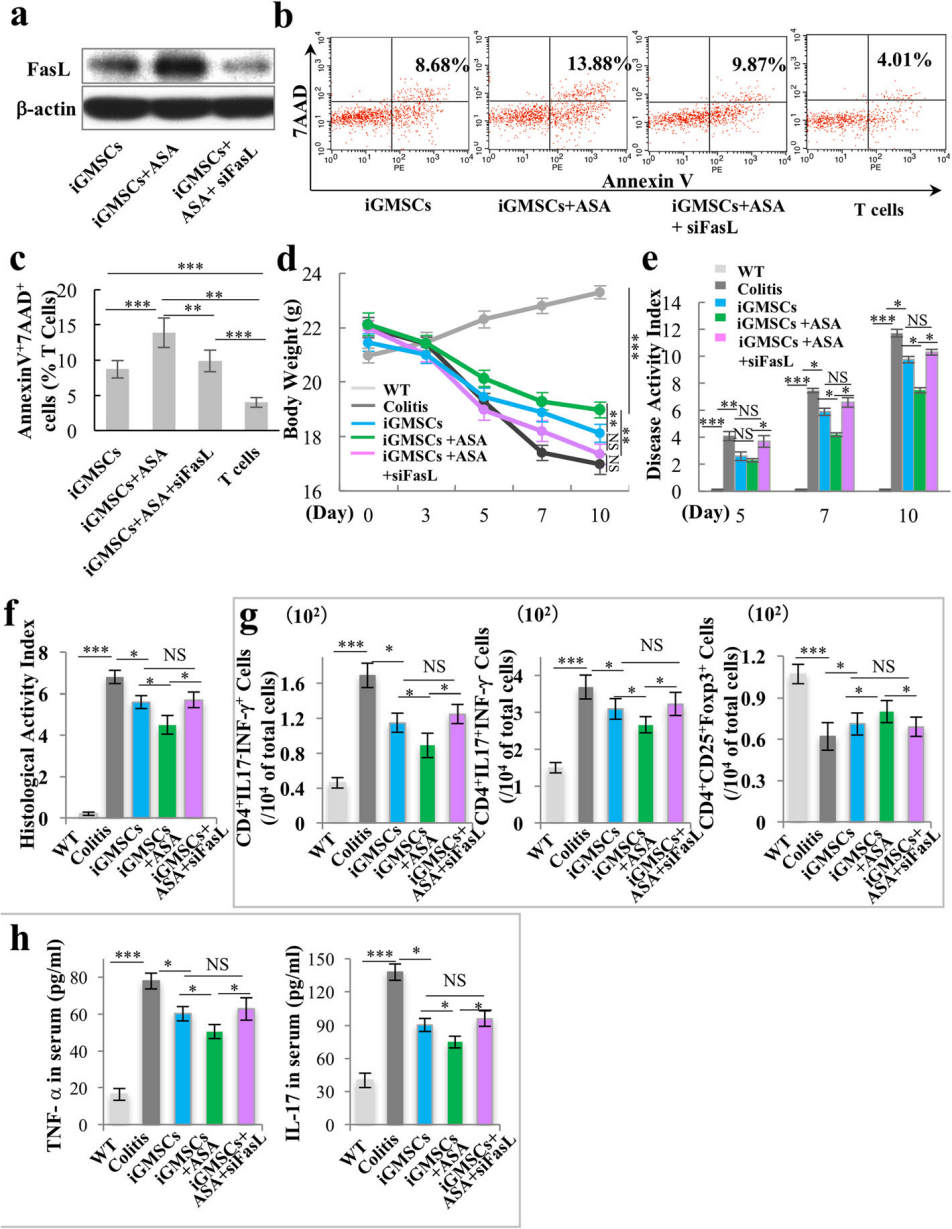
ASA treatment rescues the impaired immunomodulation capacity of iGMSCs via upregulation of FasL. **a** Western blot analysis showed that FasL siRNA downregulated the expression level of FasL in ASA-iGMSCs. **b**, **c** In vitro co-culture system showed that FasL siRNA-treated ASA-iGMSCs was not able to sufficiently induce Annexin V^+^/7AAD^+^ T cell apoptosis compared with untreated ASA-iGMSCs. **d**–**f** FasL siRNA-treated ASA-iGMSCs showed decreased immunomodulation capacity compared with untreated ASA-iGMSCs, as assessed by **d** amelioration of losing body weight, **e** decreased disease activity index (DAI), and **f** alleviation of colitis histologic activity index (HAI). **g** FasL siRNA treatment inhibits ASA-iGMSCs’ ability to reduce the levels of Th1 and Th17 cells and elevate the levels of Treg cells in colitis mice. **h** FasL siRNA treatment inhibits ASA-iGMSCs’ ability to downregulate the serum levels of TNF-α and IL-17 in colitis mice. *n* = 5 in each group. Scale bar = 200 μm. *P < 0.05. **P < 0.01. ***P < 0.005. Error bars: mean ± SD

To further confirm if ASA rescued the immunomodulation properties of iGMSCs through upregulating FasL, we knockdown FasL by using siRNA in ASA-iGMSCs (Fig. 4a and Additional file 1: Figure S5a). The co-culture system showed that downregulating FasL resulted in a decreased capacity to induce both the early (Annexin V^+^/7AAD^−^ cells) and later (Annexin V^+^/7AAD^+^ cells) T cell apoptosis (Fig. 4b, c and Additional file 1: Figure S5b) when compared with ASA-iGMSCs.

Then, we confirmed the functional role of FasL in ASA-associated immunomodulation in vivo. We systemically infused GMSCs, ASA-iGMSCs, and ASA-treated FasL-knockdown iGMSCs into colitis mice and found that ASA was not able to rescue the therapeutic effects of iGMSCs with si-FasL treatment, the phenotype of which displayed decreased body weight, exacerbated colonic inflammation, increased DAI, and recovered colitis HAI, compared to the iGMSC-treated group (Fig. 4d–f). Moreover, ASA also failed to rescue the capacity in siFasL-treated iGMSCs to downregulate the level of Th1 and Th17 cells and upregulate the level of Tregs by flow cytometric analysis (Fig. 4g). Furthermore, ASA treatment was not able to decrease the level of TNF-α and IL-17 in colitis mice serum after inhibition of FasL in iGMSCs (Fig. 4h).

Taken together, these data demonstrated that ASA treatment promoted the immunomodulation properties of iGMSCs via the FasL pathway.

## Discussion

It has been more than 50 years since the first cell therapy was successfully performed [18]. More recently, systemic infusion of MSCs has been used to treat a variety of autoimmune and other diseases [19–22]. MSCs from dental origins have emerged as a promising cell source for the treatment of autoimmune diseases due to their extensive immunomodulatory properties [8, 14, 23].

GMSCs are MSCs of dental origin and readily accessible; gingival tissues have the advantage of scarless wound healing. Moreover, GMSCs are one of the MSCs derived from the neural crest and have favorable capacity to regulate immune responses [3]. However, obtaining healthy gingival tissues could be a challenge, since inflammation is commonly encountered in the clinical situation. Previous studies have identified MSC-like cells isolated from inflamed gingiva (iGMSCs) and demonstrated their multilineage differentiation properties [9]. However, little is known whether the inflammation microenvironment would cause alteration of GMSCs’ immunomodulation capacity. In the present study, we revealed that iGMSCs obtained decreased stemness and displayed significant deficiency of immunomodulation properties when compared with control GMSCs. We revealed that iGMSCs might be differentiated into a second cell population by the effects of inflammatory cytokines and other factors. This second population then loses the MSC antigens together with FasL expression. ASA, by avoiding inflammation, impedes this differentiation.

Multiple regulators have been proposed to demonstrate the therapeutic effect of MSCs, such as transforming growth factor β (TGF-β), nitic oxide (NO), interleukin-10 (IL-10), and telomerase reverse transcriptase (TERT) [24–26]. The Fas/FasL pathway, which represents a typical mechanism of apoptotic signaling in many cell types, was found to play an important functional role of receptor/ligand in cell-based therapies [16, 27]. Activated T cells expressed an elevated level of Fas, and MSCs were found to express FasL [28]. Previous studies have indicated that FasL was involved in the GMSC-mediated therapeutic effect [8]. The possible mechanism may be due to FAS-regulated monocyte chemotactic protein 1 (MCP-1) secretion by MSCs, which could recruit T cells for FasL-mediated apoptosis. The apoptotic T cells would subsequently trigger macrophage producing high levels of TGF-β, which in turn leads to the upregulation of CD4^+^CD25^+^Foxp3^+^ regulatory T cells so as to induce immune tolerance [27]. In the present study, we demonstrated that the expression of FasL in iGMSCs was decreased, which leads to less effective ability to induce T cell apoptosis and results in less therapeutic efficacy to cure colitis mice. Then, we try to find ways to recover the therapeutic effects of iGMSCs. Previous studies promoted the immunomodulatory capacity of MSCs by genetic modification or pretreating MSCs with cytokines [29, 30]. Though these methods have shown substantial therapeutic effects, their safety still remains to be a concern for both patients and practitioners. ASA, commonly known as aspirin, is one of the widely used NSAIDs to relieve pain and prevent adverse cardiovascular event. Recently, other therapeutic potentials of ASA were discovered, such as prevention of cancer and reducing the risk of preterm preeclampsia [31, 32].

It was reported that ASA is able to promote MSC osteogenesis and bone regeneration [33, 34] but inhibits MSC proliferation [35]. Recent studies also revealed that ASA could enhance the immunomodulatory capacity of MSCs, which eventually results in immune tolerance [14, 36]. Compared with the untreated iGMSCs, ASA treatment rescued the therapeutic effects of iGMSCs in curing colitis mice, leading to downregulation of Th1 and Th17 cells and upregulation of T cell apoptosis and Treg cells. Also, serum TNF-α and IL-17 levels were downregulated. Thus, we confirmed that ASA treatment successfully rescued the impaired immunomodulation properties of iGMSCs. Mechanistically, we demonstrated that ASA treatment elevated the expression of FasL in iGMSCs, while ASA treatment was unable to rescue the immunomodulatory capacity in iGMSCs with FasL knockdown tested in vitro or in vivo*.*

Though we revealed ASA was able to rescue the therapeutic ability of MSCs from inflamed microenvironment through the Fas/FasL pathway, it may only represent one of the multiple mechanisms of MSC-based therapy. The detailed molecular and cellular mechanisms still remain to be elucidated. ASA represents a small-molecule drug with easy access, which has been proved to be safe in long-term clinical application. Further studies are needed to explore effective means such as ASA to enhance the promising therapeutic effects of MSC-based treatment.

## Conclusions

In the present study, we demonstrated that iGMSCs showed compromised stemness and deficient immunomodulation capacity. ASA treatment improves the immunomodulatory capacities of iGMSCs by elevating FasL. ASA treatment may be an efficient and potential pharmacologic approach to rescue the therapeutic effects of iGMSCs.

## Supplementary information

**Additional file 1:** **Figure S1.** iGMSCs exhibited a second population in CD90, CD105, and CD73, as assessed by flow cytometry analysis. **Figure S2.** iGMSCs exhibited decreased osteogenic, adipogenic, and chondrogenic differentiation potential. **a** The mineralized nodule formation in GMSCs and iGMSCs as assessed by alizarin red staining. **b** The lipid droplet formation in GMSCs and iGMSCs as assessed by Oil-red O staining. **c** Chondrogenic differentiation of GMSCs and iGMSCs as assessed by toluidine blue. *n* = 5 in each group. Scale bar, 100 μm. **P < 0.01. Error bars: mean ± SD. **Figure S3.** Related to Fig. 2**:** iGMSCs induced less Annexin V^+^7AAD^−^ T cell apoptosis compared with control GMSCs, as assessed by flow cytometry. *n* = 5 in each group. **P < 0.01. Error bars: mean ± SD. **Figure S4.** Related to Fig. 3: a iGMSCs induced less Annexin V^+^7AAD^−^ T cell apoptosis compared with control GMSCs, and ASA treatment elevated Annexin V^+^7AAD^−^ T cell apoptosis induced by iGMSCs, as assessed by flow cytometry. **b** The quantification of protein expression level according to Fig.3b. **c** The expression levels of iNOS, PEG6, and TGFβ in iGMSCs and GMSCs, as assessed by qPCR. **d** siFasL siRNA knockdown efficiency in GMSCs was shown by Western blotting. **e** In vitro coculture system showed a significantly decreased capacity of siFasL-treated GMSCs to induce AnnexinV^+^7AAD^+^ T cells apoptosis when compared with the GMSC group. *n* = 5 in each group. *P < 0.05. **P < 0.01. ***P < 0.005. Error bars: mean ± SD. **Figure S5.** Related to Fig. 4: a The quantification of protein expression level according to Fig.4a. **b** ASA-iGMSCs induced more Annexin V^+^7AAD^−^ T cell apoptosis compared with iGMSCs, and siFasL treatment inhibited Annexin V^+^7AAD^−^ T cell apoptosis induced by ASA-iGMSCs, as assessed by flow cytometry. *n* = 5 in each group. *P < 0.05. **P < 0.01. Error bars: mean ± SD.

## Data Availability

The authors confirm that all data generated or analyzed during this study are available.

## Abbreviations

GMSCs: Gingiva-derived mesenchymal stem cells
iGMSCs: Gingiva-derived mesenchymal stem cells from inflamed gingival tissues
BMMSCs: Bone marrow mesenchymal stem cells
FasL: Fas ligand
DSS: Dextran sulfate sodium
ASA: Acetylsalicylic acid
Tregs: Regulatory T cells
