# Supplementary material for: Acetylsalicylic acid rescues the immunomodulation of inflamed gingiva-derived mesenchymal stem cells via upregulating FasL in mice
Source: Stem Cell Res Ther. 2019 Dec 3;10:368. doi: 10.1186/s13287-019-1485-5 (PMC6892130; doi:10.1186/s13287-019-1485-5)
Supplement: Supplementary file 1 — Additional file 1: Figure S1. iGMSCs exhibited a second population in CD90, CD105, and CD73, as assessed by flow cytometry analysis. Figure S2. iGMSCs exhibited decreased osteogenic, adipogenic, and chondrogenic differentiation potential. a The mineralized nodule formation in GMSCs and iGMSCs as assessed by alizarin red staining. b The lipid droplet formation in GMSCs and iGMSCs as assessed by Oil-red O staining. c Chondrogenic differentiation of GMSCs and iGMSCs as assessed by toluidine blue. n = 5 in each group. Scale bar, 100 μm. **P < 0.01. Error bars: mean ± SD. Figure S3. Related to Fig. 2: iGMSCs induced less Annexin V+7AAD− T cell apoptosis compared with control GMSCs, as assessed by flow cytometry. n = 5 in each group. **P < 0.01. Error bars: mean ± SD. Figure S4. Related to Fig. 3: a iGMSCs induced less Annexin V+7AAD− T cell apoptosis compared with control GMSCs, and ASA treatment elevated Annexin V+7AAD− T cell apoptosis induced by iGMSCs, as assessed by flow cytometry. b The quantification of protein expression level according to Fig.3b. c The expression levels of iNOS, PEG6, and TGFβ in iGMSCs and GMSCs, as assessed by qPCR. d siFasL siRNA knockdown efficiency in GMSCs was shown by Western blotting. e In vitro coculture system showed a significantly decreased capacity of siFasL-treated GMSCs to induce AnnexinV+7AAD+ T cells apoptosis when compared with the GMSC group. n = 5 in each group. *P < 0.05. **P < 0.01. ***P < 0.005. Error bars: mean ± SD. Figure S5. Related to Fig. 4: a The quantification of protein expression level according to Fig.4a. b ASA-iGMSCs induced more Annexin V+7AAD− T cell apoptosis compared with iGMSCs, and siFasL treatment inhibited Annexin V+7AAD− T cell apoptosis induced by ASA-iGMSCs, as assessed by flow cytometry. n = 5 in each group. *P < 0.05. **P < 0.01. Error bars: mean ± SD. [file 13287_2019_1485_MOESM1_ESM.docx]

**Additional file 1**


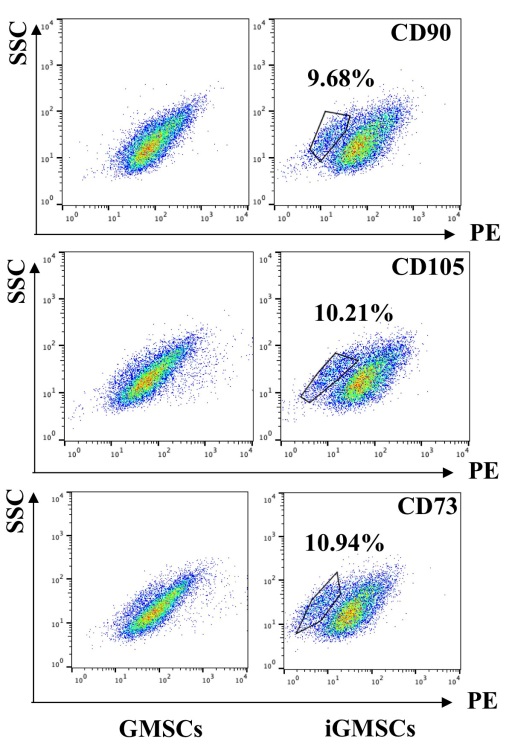


**Figure S1** iGMSCs exhibited a second population in CD90, CD105, and CD73, as assessed by flow cytometry analysis.


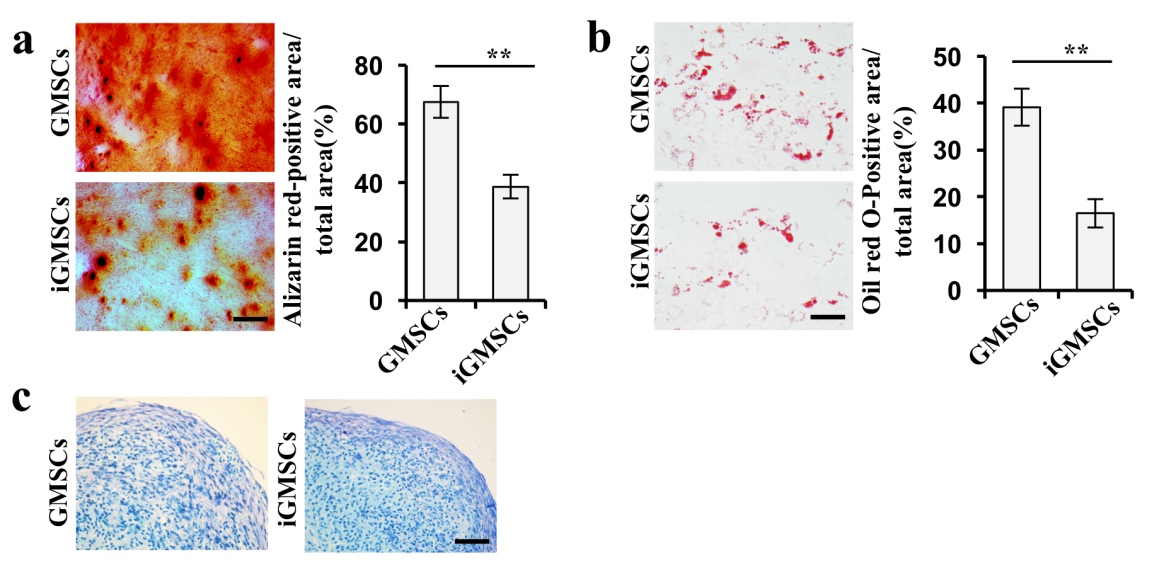


**Figure S2** iGMSCs exhibited decreased osteogenic, adipogenic, and chondrogenic differentiation potential. **a** The mineralized nodule formation in GMSCs and iGMSCs as assessed by alizarin red staining. **b** The lipid droplet formation in GMSCs and iGMSCs as assessed by Oil-red O staining. **c** Chondrogenic differentiation of GMSCs and iGMSCs as assessed by toluidine blue. *n* = 5 in each group. Scale bar, 100 μm. **P < 0.01. Error bars: mean ± SD.


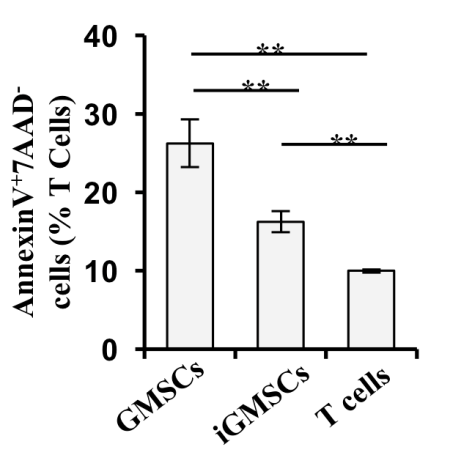


**Figure S3.** **Related to Figure 2:** iGMSCs induced less Annexin V^+^7AAD^-^ T cell apoptosis compared with control GMSCs, as assessed by flow cytometry. *n* = 5 in each group. **P < 0.01. Error bars: mean ± SD.


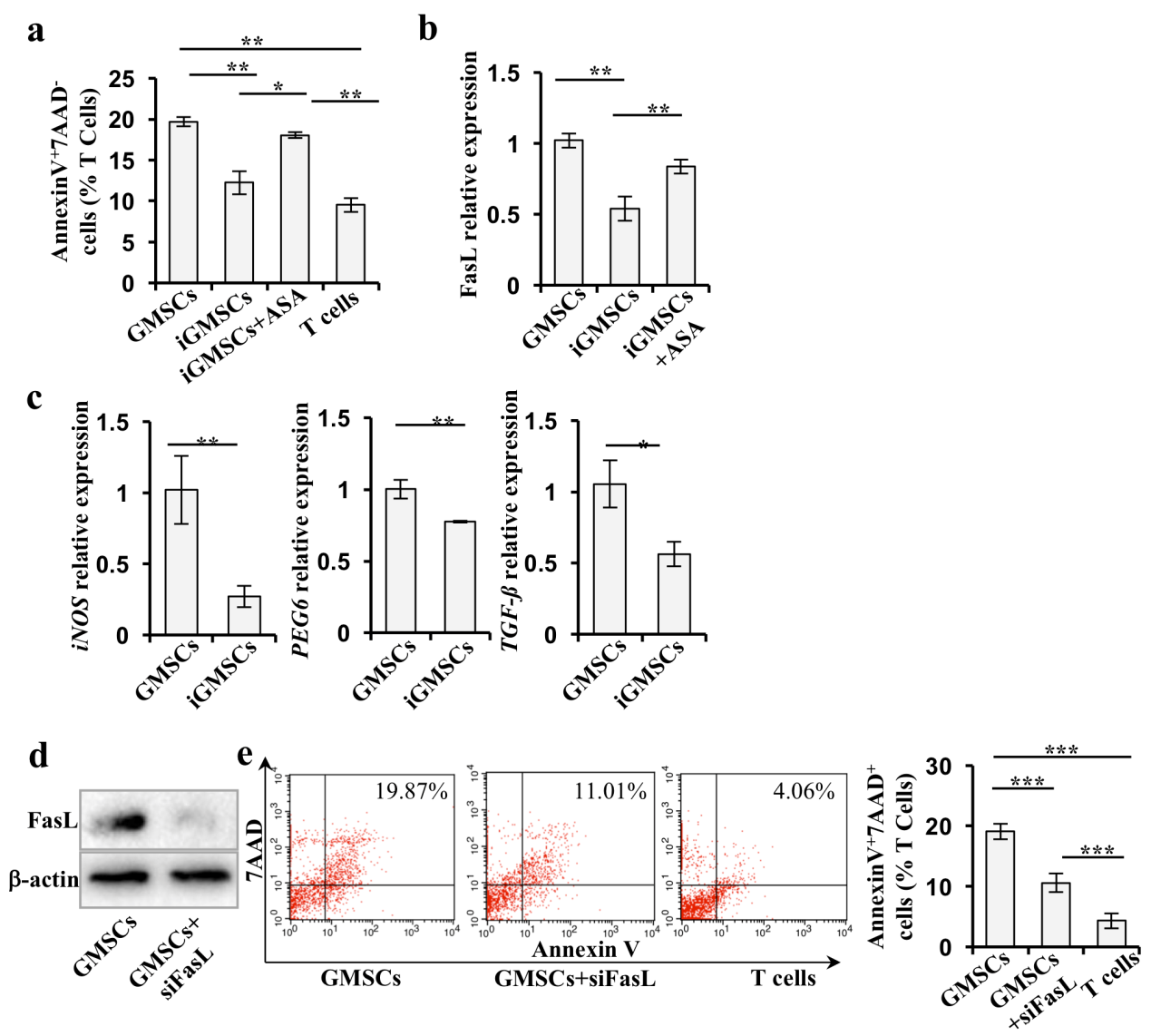


**Figure S4. Related to Figure 3: a** iGMSCs induced less Annexin V^+^7AAD^-^ T cell apoptosis compared with control GMSCs, and ASA treatment elevated Annexin V^+^7AAD^-^ T cell apoptosis induced by iGMSCs, as assessed by flow cytometry. **b** The quantification of protein expression level according to Fig.3b. **c** The expression levels of iNOS, PEG6, and TGFβ in iGMSCs and GMSCs, as assessed by qPCR. **d** siFasL siRNA knockdown efficiency in GMSCs was shown by Western blotting. **e** In vitro coculture system showed a significantly decreased capacity of siFasL-treated GMSCs to induce AnnexinV^+^7AAD^+^ T cells apoptosis when compared with the GMSC group. *n* = 5 in each group. *P < 0.05. **P < 0.01. ***P < 0.005. Error bars: mean ± SD.


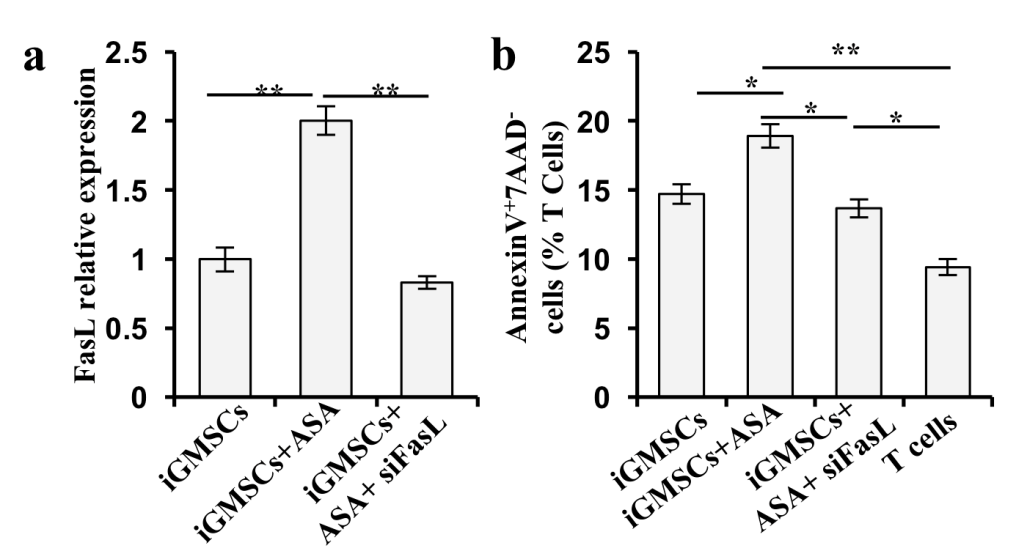


**Figure S5. Related to Figure 4: a** The quantification of protein expression level according to Fig.4a. **b** ASA-iGMSCs induced more Annexin V^+^7AAD^-^ T cell apoptosis compared with iGMSCs, and siFasL treatment inhibited Annexin V^+^7AAD^-^ T cell apoptosis induced by ASA-iGMSCs, as assessed by flow cytometry. *n* = 5 in each group. *P < 0.05. **P < 0.01. Error bars: mean ± SD.
